# 

**Published:** 1859-11

**Authors:** 


### NOTICE EXTRAORDINARY.

Subscribers will please remember that all subscriptions expire with the December number, and that in no single instance is our Journal sent without being ordered and paid for, so that those who do not desire to take it another year, need not be at the trouble and expense of writing to that effect. The editor studies how to keep.in good health, and succeeds pretty well, having taken but one remembered dose of medicine in twenty years, and that was one of his own pills, and it not only cured him, but he has remained cured, has never lost a meal for want of an appetite, nor a night's sleep from sickness. One of our ways of keeping well is to keep in a good humor, to study how to secure a succession of pleasurable feelings, and how to avoid all jarrings, even of a slight character. Hence the request above—for consider what a jar it would be to open a letter, and instead of finding a dollar, find a discontinuance! But what a succession of pleasurable feelings to find old subscribers renewing, not only their remittances of money, but their expressions of good will and satisfaction, and as a proof of the same, sending the names of new subscribers, obtained through their personal influence. While on the subject, we may name also, that another means of avoiding "disagreeables" is, when the first word or line of a letter indicates ill temper, not to throw it in the grate, but to close it at once, and lay it carefully away, to make "coals of fire," at some subsequent opportunity, which, by the way, is pretty sure to occur; and really it is a kind of a pleasure sometimes to "heap" them up sky high!

Another way of ours is, whenever a letter is seen to be over a page, we turn to the writer's name, or the few last lines, and determine by them whether to take any notice of it or not. We cannot afford to read interminable letters, unless they are from our actual patients; them we read with interest and care, because we are paid for it, and are anxious to know all about them.

Subscribers are requested to look at one of the advertising pages, and read the contents of the first five numbers of the Fireside Monthly. Its object is to provide a monthly reading for families which will always be safe, practical, and pertinent to the times; a publication which aims never to offend the religious sentiment of any evangelical christian; a publication which, thou h not professedly religious, shall never be against religion, the Bible, or the Sabbath day. It is purposed to exclude fictitious reading, and confine its pages, at least for the present, to fact, to the great realities of life. We know of no similar publication in the Union. Such an one is needed, but we are doubtful about its being sustained. The useful and the true is not now largely sought for; fiction and frivollty is the fashion of the times, even in many excellent families. We have risked something, and are willing to lose something for the bare chance of founding a publication which may be antagonistic to the pernicious influences of much of our periodical literature. If any of our subscribers will lend their aid in our enterprise by subscribing to the "Fireside Monthly," which is one dollar and fifty cents a year, we will offer the inducement that two dollars sent previous to January 1st, 1860, will pay for the Journal of Health aud the Fireside Monthly for one year from that date, and we are persuaded that a physical and moral result will be observed, which many times the money expended in ordinary ways would fail to secure, for the Journal and Fireside labor to save the life, and soul, and secure the health of both body and mind.

☞Please address all subscriptions for the year 1860, (sending fractions of a dollar in postage stamps) to "HALL'S JOURNAL OF HEALTH BOX, NEW YORK.

